# Optimization of *EGFR* mutation detection by the fully-automated qPCR-based Idylla system on tumor tissue from patients with non-small cell lung cancer

**DOI:** 10.18632/oncotarget.21476

**Published:** 2017-10-04

**Authors:** Marius Ilie, Catherine Butori, Sandra Lassalle, Simon Heeke, Nicolas Piton, Jean-Christophe Sabourin, Virginie Tanga, Kevin Washetine, Elodie Long-Mira, Priscilla Maitre, Nathalie Yazbeck, Olivier Bordone, Virginie Lespinet, Sylvie Leroy, Charlotte Cohen, Jérôme Mouroux, Charles Hugo Marquette, Véronique Hofman, Paul Hofman

**Affiliations:** ^1^ Laboratory of Clinical and Experimental Pathology, Pasteur Hospital, FHU OncoAge, University Côte d’Azur, Nice, France; ^2^ IRCAN Inserm U1081/CNRS 7284, FHU OncoAge, University Côte d’Azur, Nice, France; ^3^ Hospital-related Biobank (BB-0033-00025), Pasteur Hospital, FHU OncoAge, University Côte d’Azur, Nice, France; ^4^ Department of Pathology, Rouen University Hospital, Rouen, France; ^5^ Department of Pulmonary Medicine and Thoracic Oncology, Pasteur Hospital, FHU OncoAge, University Côte d’Azur, Nice, France; ^6^ Department of Thoracic Surgery, Pasteur Hospital, FHU OncoAge, University Côte d’Azur, Nice, France

**Keywords:** NSCLC, EGFR, targeted therapy, optimization, PCR

## Abstract

Treatment with EGFR inhibitors is limited to patients with advanced/metastatic non-small cell lung cancer who have known *EGFR* mutations. Currently, patient care has to respond to several imperatives to make these inhibitors broadly available to all patients; fast and accurate detection of *EGFR* mutations by a sensitive and specific standardized cost-effective method, easy-to-implement in settings with limited expertise in molecular diagnostics.

We evaluated the Idylla™ EGFR Mutation Assay (Biocartis) for the detection of *EGFR* mutations in archived formalin-fixed paraffin-embedded (FFPE) tumor samples from a series of 55 patients with lung adenocarcinoma and compared these results with those obtained by a pyrosequencing ISO-15189 accredited laboratory method. The comparison was made on both whole surgical tumor sections and on three artificially constructed small biopsies (∼1 mm) from the same FFPE blocks. Cost-effectiveness and turnaround time comparison between the two methods was performed.

On both whole tissue sections and on biopsy cores, the Idylla™ and pyrosequencing had an agreement of 95% (52/55). The Idylla™ *EGFR* Assay produced results faster and more cost-effective than pyrosequencing.

The Idylla™ system showed a good sensitivity and was cost-saving in our setting. Because of the easy workflow, the Idylla™ system has the potential to expand EGFR testing to more pathology laboratories in a reliable and fast manner.

## INTRODUCTION

The detection of *EGFR* activating or resistance mutations associated with advanced or metastatic non-small cell lung cancer (NSCLC) allows targeted treatment with first-, second- or third-generation tyrosine kinase inhibitors [1, 2]. Several constraints quickly emerged from this research: (i) tissue samples are increasingly small in size due to the less and less invasive sampling techniques, (ii) the time-to-result must be faster to fulfill clinical needs, leading also to competition between the different laboratories and the different institutions, and (iii) support and reimbursement of these tests, whose number of requests is increasing rapidly, create a new economic issue [1, 3–6]. There are many molecular biological methods deployed for detection of EGFR activating or resistance mutations but with various and different sensitivity [7–9]. These methods have variable cost, and results turn-around time depends on their complexity and the sample flow. They require technical and medical expertise, especially for results analysis.

The aim of this study is to analyze benefits and disadvantages of the Idylla™ system for the detection of *EGFR* mutations in a series of 18 NSCLC patients whose mutated status was already known by pyrosequencing, which is our reference method in the laboratory, and accredited according to the ISO 15189 standard (http://www.cofrac.fr/en/organismes/fiche.php?entite_id=82017619). This comparison focusses on sensitivity, turnaround time, and cost of both methods used (Idylla™ *versus* pyrosequencing).

## RESULTS

### Analytical sensitivity

In the first assessment on whole tissue sections, Idylla™ demonstrated agreement with the routine reference method in 52 of the 55 cases (95%), with a sensitivity of 100%, specificity of 82.35%, positive predictive value of 92.68% and negative predictive value of 100%. In two samples, Idylla™ detected the *EGFR* mutation observed by the reference method, as well as a supplementary mutation L861Q on exon 21 and T790M mutation on exon 20 not detected by the reference assay (Cases 1 and 25, Supplementary Table 1). In one sample with *EGFR* wild-type status by pyrosequencing, the Idylla™ system detected a T790 mutation on exon 20 (Case 37, Supplementary Table 1).

In the second assessment on the tissue microarrays (TMA) biopsy cores, Idylla™ demonstrated agreement with the reference method in 52 of the 55 cases (95%), with a sensitivity of 100%, specificity of 85%, positive predictive value of 92.11% and negative predictive value of 100%.

In three cases, the pyrosequencing method failed to detect the *EGFR* mutations on TMA biopsy cores (% tumor cells, range 10 to 40%), whereas the analysis on the corresponding whole tissue sections having more than 50% of tumor cells demonstrated the same mutations by the Idylla system (Cases 4, 25 and 37; Supplementary Table 1). Moreover, an immunohistochemistry analysis on the biopsy cores with the monoclonal antibodies against EGFR del19 or L858R mutated proteins showed high expression for the mutated corresponding protein (Supplementary Figure 1) [10].

### Turnaround times

For the analysis by pyrosequencing (either on whole sections or from biopsies), the average time from sample to result is about 12 hours. Turnaround time includes microtome cutting of 3 tissue sections, DNA extraction and sequencing, and final analysis.

For the analysis by the Idylla™ system (either on whole sections or from biopsies), the average time from sample to result is about 3 hours. Turnaround time includes microtome cutting, and fully integrated DNA preparation, qPCR, and automated analysis.

### Cost analysis

A cost analysis has been done in our laboratory. All costs (except equipment acquisition and maintenance) have been included in this overview. For pyrosequencing total cost is divided as following: close to 57% of total cost is related to Hands on time and 39% to reagent kit cost. The remain part is related to extraction and some consumables i.e. 4.5% of total cost. For Idylla and by design, the outcome is different. 99.4% of the total cost is related to cartridge price as it includes all reagents needed for liquefaction, DNA extraction, Amplification and fluorescence measurement. Hand on time is 0.5% of total cost of the test. The cost analysis shows up a difference of 38 EUR in favor of Idylla™ vs Pyrosequencing for EGFR mutation analysis.

### Coverage of EGFR mutation sequences

The different *EGFR* mutations present in the Qiagen EGFR kit and the Idylla™ EGFR Mutation Assay are available on the websites of these two companies (https://www.qiagen.com/us/resources/technologies/oncology-companion-diagnostics/therascreen-egfr-test-usa-labs/ and https://media.biocartis.com/biocartis/documents/TECH_FICHE-EGFR-RUO-01122016.pdf).

The Idylla™ EGFR Mutation Assay (Biocartis) detects 51 mutations and indels *versus* the Therascreen EGFR Pyro Kit (Qiagen) that detects 21 mutations and indels. The *EGFR* mutations detected by the pyrosequencing method and tested on Idylla™ system in this study are listed in Table 1 and Supplementary Tables 1 and 2.

**Table 1:** Main clinical and biological data from patients included in the study

Patients demographics	*n* = 55
**Age**	
Median (range)	64.5 (45–84)
**Sex**	
Male Female	42 (76%) 13 (24%)
**Tobacco Use History**	
Former or Current smoker Never smoked	4 (7%) 51 (93%)
**Tumor cell content (%)**	
20% 30% 40% 50% 60% 70% 80%	4 (7%) 11 (20%) 4 (7%) 14 (25%) 1 (2%) 8 (15%) 13 (24%)
**Stage**	
I II III IV	3 (5%) 4 (7%) 2 (4%) 46 (84%)
EGFR **mutation status** (reference assay ISO 15189)	
** Wild-type** ** Exon 18** p.G719A **Exon 19** p.Glu746_Ala750del p.Glu746_Ser752delinsVal p.Leu747_Ala750delinsPro p.Leu747_Pro753delinsSer ** Exon 20** ** Exon 21** L858R L861Q ** Double mutations** p.Leu747_Pro753delinsSer + T790M p.Glu746_Ala750del + T790M G719C + S768I G719S + S768I L858R + T790M	15 (27%) 2 (4%) 12 (22%) 8 1 1 2 1 (2%) 18 (33%) 17 1 7 (13%) 1 3 1 1 1

## DISCUSSION

Optimization of the management of patients having lung cancer requires fast molecular analysis results, in particular the *EGFR* activating or resistance mutations analysis (http://www.e-cancer.fr/content/download/63175/568709/file/OUTTHERANOS10.pdf) [6]. Different methods having variable sensitivity, ranging from 0.001% to 20%, are possible for this detection [7–9]. Specificity of these methods may vary as well. Contamination of oligonucleotides, particularly with highly sensitive methods, can sometimes occur [11]. Our results show that the detection of *EGFR* mutations by Idylla™ is as sensitive as the pyrosequencing method which is accredited in our laboratory since 2013. Interestingly, the Idylla™ system outperformed the standard pyrosequencing, which missed mutations in three cases on both whole tissue tumor and TMA biopsies. At the same time, the presence of these mutations was confirmed by an immunohistochemical analysis thanks to two antibodies specifically directed against the del19 or L858R mutated proteins (Supplementary Figure 1) [10]. Moreover, with the detection of the T790M mutation becoming essential for the treatment with osimertinib [12], it is interesting to note that the Idylla™ system was able to detect two supplementary T790M mutations not being detected by the reference method. Thus, the limit of detection (LOD) of the T790M mutation by Idylla was below 10.7% allelic frequency (reported LOD for the EGFR *therascreen* Pyro kit) in two specimens with 50% tumor cells.

Comparative analysis of *EGFR* mutation panels, present on the Idylla™ and Qiagen Therascreen Pyro kit, showed that 27 activating mutations were not covered by the Qiagen Therascreen Pyro kit. In this study we showed that the Idylla™ approach is much faster (Time to results in a few hours) and the cost is comparable if we exclude the technician time which is significantly more for the pyrosequencing test than for Idylla which requires less than five minutes of hands on time. The Idylla™ method requires a smaller amount of fixed tissue to be loaded in the cartridge than for the pyrosequencing method (1 tissue section versus 3 sections). Even if it is a rather subjective criterion, the Idylla™ method is highly appreciated by the technical and medical team because it does not require high expertise in molecular biology for the hands-on time of the test, neither for results interpretation.

If hotspot mutation analysis and next generation sequencing (NGS) approaches are currently competing, we have to acknowledge the immediate need for clinicians to rapidly treat a patient suffering from metastatic NSCLC with molecules that have been authorized for treatment beyond the scope of including these patients in therapeutic trials for highly specialized centers [6, 13]. In this context, it is important to consider the best algorithm leading to rapid and reliable results of *EGFR* mutation status, which are the only mutations allowing the administration of targeted therapies (first, second and third generation TKI inhibitors) [9, 14]. *EGFR* mutation analysis in patients with advanced or metastatic non-epidermoid pulmonary carcinoma has become a systematic and mandatory clinical practice [11]. Although frequency of these mutations is only seen in 10–15% of adenocarcinomas in Caucasian patients, all of them may benefit from these targeted therapies. Other mutation analyses (particularly via NGS), for instance in patients not having EGFR mutation, can be used to include patients in clinical trials [15, 16].

*EGFR* gene mutation results should be obtained quickly, given the rapid tumor progression and the availability of effective targeted therapy. Thus, clinicians are urging laboratories to develop rapid technological approaches [6]. One of the bottlenecks observed for short turnaround time results is the organization of a large number of pathology laboratories working independently from the molecular biology laboratories to which samples (extracted DNA, non-stained slides, tissue slides or tissue blocks) are sent for *EGFR* mutation analysis [17, 18]. Although these laboratories (especially general hospitals) might be geographically close to each other, sample transmission by pathologists must always be as quick as possible [17]. Not working physically in the same laboratory imposes a rigorous traceability which, if failed, could be a potential source of error due to the distance between the labs. In this context, the search for *EGFR* mutation status by the Idylla™ method combines two advantages: i) the short turnaround time (result in less than three hours) thanks to the integration of DNA preparation, qPCR and analysis combined in one cartridge on the same system; and (ii) the tissue section obtained within the pathology laboratory that can be loaded into the Idylla™ cartridge immediately and can be run by the Idylla™ system (Table 2) in the same laboratory [19–22]. In theory, all pathology laboratories can therefore use this method as long as a pathologist confirms the presence of a tumor zone before performing the tissue cut. Methods used to determine the mutational status of *EGFR* are more and more sensitive with in addition the requirement to be specific [11]. This sensitivity must take into account, beyond the quality, the amount of extracted DNA, which is becoming increasingly challenging due to the reduced biological sample available and association with less and less invasive or imaging-guided sampling methods [23, 24]. In our study we compared for the same patients and using the same initial tissue blocks, the sensitivity of the Idylla™ method with whole tissue sections and with tissue sections made from biopsies built on pre-selected areas with the initial tissue blocks. These “artificial” biopsies can thus be compared, based on their size, to bronchial biopsies that would be obtained during bronchial endoscopy or during a transthoracic biopsy. The concordance on these different sample types was excellent, confirming the high sensitivity of Idylla™ method also on biopsy samples which represent the vast majority of sample types in the laboratories for the detection of *EGFR* mutations [19]. The mean percentage of tumor cells present in the areas leading to DNA extraction of whole-cut tissue was 53% (range, 20% to 80%), and on the corresponding biopsies of 45% (range, 5% to 80%). In the future, performance of Idylla™ on lower percentage of tumor cells should be tested. It should be noted that most of the other techniques used for *EGFR* mutations detection (except ddPCR) require a percentage of tumor cells at least greater than 20% [25].

**Table 2:** Advantages and disadvantages of Idylla™ versus pyrosequencing for *EGFR* mutation detection

Criteria	Idylla™	Pyrosequencing
*Tissue saving (one section)*	+	–
*Amount of tissue material*	+	–
*Extraction time and analysis*	+	–
*Mutation panel*	+	+
*Mutation coverage*	+	–
*Ease of use and interpretation*	+	–
*Interpretation of the results*	+	–
*Time to results (< 3 h)*	+	–
*Sensitivity and specificity*	+	+
*DNA availability for additional testing*	–	+
*Cost*	+ (less expensive)	– (More expensive)

One of the advantages of the Idylla™ system is that it requires only one 10 μm thick FFPE tissue section, as opposed to the pyrosequencing method for which DNA extraction within our laboratory requires a minimum of three tissue sections of 10 μm. This is now really important for NSCLC biopsy specimens, with the high number of biomarkers to be evaluated and for which tissue material saving is a key element [16]. Immunotherapy development will probably require multiple immunohistochemical studies combined with the search for several biomarkers being predictive of a therapeutic response [26, 27]. This could lead to the establishment of new algorithms for tissue sample management in order to search all potential targets (protein or genetic) for personalized treatments [27]. One limitation of the Idylla™ system is that it is not possible to obtain the residual DNA extracted after *EGFR* gene analysis in the cartridge (Table 2). Extracting DNA upfront for pyrosequencing analysis allows (if enough DNA amount) to perform broader mutational analysis like by NGS, and look for other genetic abnormalities which are potentially accessible to a targeted therapy in the case no mutation is observed for *EGF*R. In this context, the need to extract DNA from a new tissue section when no *EGFR* mutations have been detected by the Idylla™ system, leads to an increase of delay of NGS results, consumption of available samples and DNA extraction costs. Still, when no tumor tissue is available, the Idylla assay have the potential to be extended to liquid biopsy which demonstrated acceptable concordance with standard testing of tumor tissue [28].

To conclude, the use of the Idylla™ system in assessing *EGFR* mutational status from paraffin-embedded tissues is a sensitive approach, allowing results within a few hours, and easily applicable to all pathology laboratories.

## MATERIALS AND METHODS

Fifty-five patients who underwent surgical resection for pulmonary adenocarcinoma between 2010 and 2016 were retrospectively included in this study. These patients gave their consent to this study which was approved by the ethics committee of the Nice CHU. The tumor samples fixed in formaldehyde and included in paraffin were selected retrospectively within the biobank of the CHU of Nice (BB-0033-00025) and the Department of Pathology, Rouen University Hospital. The EGFR mutation status was known from a prospective analysis carried out systematically by a pyrosequencing method [therascreen EGFR Pyro Kit (Qiagen, Hilden, Germany)] (www.qiagen.com/fi/shop/detection-solutions/personalized-healthcare/therascreen-egfr-pyro-kit). For this analysis, 3 tissue sections of 10 μm were cut, and then DNA extraction was carried out using a DNA extraction kit (QIAamp DNA FFPE Tissue Kit, Qiagen). The different clinical, pathological and molecular parameters of samples are listed in Table 1. The *EGFR* mutations detected by the pyrosequencing method and tested on Idylla™ system are listed in Supplementary Tables 1 and 2.

The Idylla™ method was carried out on the same tissue blocks according to the protocol described above [19–22]. Briefly, a 10 µm thick FFPE tissue section is loaded by a sterile forceps directly in the cartridge then placed in the Idylla™ system allowing integrated DNA extraction and mutational hotspot analysis (Supplementary Figure 2). For this study we used the RUO Idylla ™ *EGFR* Mutation Assay (Biocartis, Mechelen, Belgium).

In a second series of analyses, three biopsies, having 1 mm diameter, were performed on each paraffin-embedded tumor block with a needle dedicated to TMA construction (Alphelys, Paris) (Supplementary Figure 2). These tissue punches were carried out on tumor zones selected by a pathologist (CB) for which the percentage of tumor cells was recorded before carrying out these three biopsies. These biopsy punches were repositioned in blocks of virgin paraffin (Supplementary Figure 2). The quality of the new biopsies was checked by a pathologist (CB) after staining with hematoxylin eosin on tissue sections made from these newly constructed blocks. The *EGFR* mutations status was then carried out by pyrosequencing method after DNA extraction from 3 sections of 10 μm and by the Idylla™ system starting directly from a 10 μm tissue section, as described above [19, 21, 25].

The following parameters were analyzed and compared for the Idylla™ system to the pyrosequencing method which is the “gold” standard technology in our lab, (EGFR accredited ISO 15189; www.cofrac.fr): sensitivity (different mutations analyzed), time to result (including DNA extraction step, analysis and sequence reading), cost per test (including extraction and analysis steps, technician time but excluding equipment acquisition and maintenance costs). The results obtained from the whole sections made on the tumor resection blocks and on the sections made on the biopsies were compared for both the pyrosequencing technique and the Idylla™ technique.

Immunohistochemistry with the two mutation-specific antibodies, one recognizing the exon 21 L858R *EGFR* mutation (pre-diluted, clone SP125, Ventana Medical Systems Inc., Tucson, AZ) and the other the E746-750 15-bp deletion in exon 19 (pre-diluted, clone SP111, Ventana), was performed as described [10]. Briefly, after deparaffinisation in xylene and rehydration through a graded series of ethanol, antigen retrieval was performed using CC1 buffer (Ventana) and the primary antibody was applied into a BenchMark Ultra automate (Ventana). For detection, the Ultraview Universal DAB detection kit (Ventana) was used. For both antibodies, positive cases were considered when ≥ 10% of neoplastic cells showed strong staining.

## SUPPLEMENTARY MATERIALS FIGURES AND TABLES






