# Supplementary material for: Optimization of EGFR mutation detection by the fully-automated qPCR-based Idylla system on tumor tissue from patients with non-small cell lung cancer
Source: Oncotarget. 2017 Oct 4;8(61):103055–62. doi: 10.18632/oncotarget.21476 (PMC5732710; doi:10.18632/oncotarget.21476)
Supplement: Supplementary file 1 [file oncotarget-08-103055-s001.pdf]

Optimization of *EGFR* mutation detection by the fully-automated qPCR-based Idylla system on tumor tissue from patients with non-small cell lung cancer

SUPPLEMENTARY MATERIALS

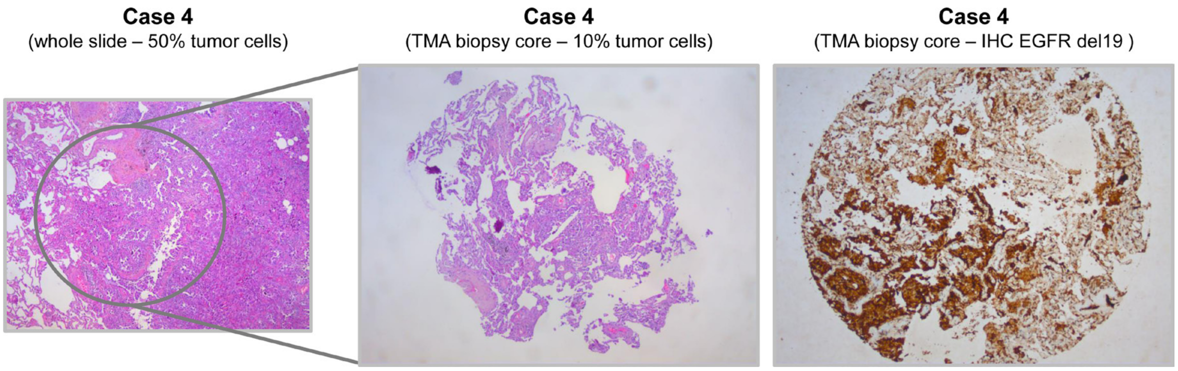

Supplementary Figure 1: Case 4 was analyzed either on whole tissue slides having 50% of tumor cells (HES staining, *left panel*), or on TMA biopsy core with 10% of tumor cells (HES staining, *middle panel*) by both pyrosequencing and Idylla™, as well as an immunohistochemistry (IHC) analysis with the mAb against EGFR del19 *(right panel)* on the TMA biopsy core.

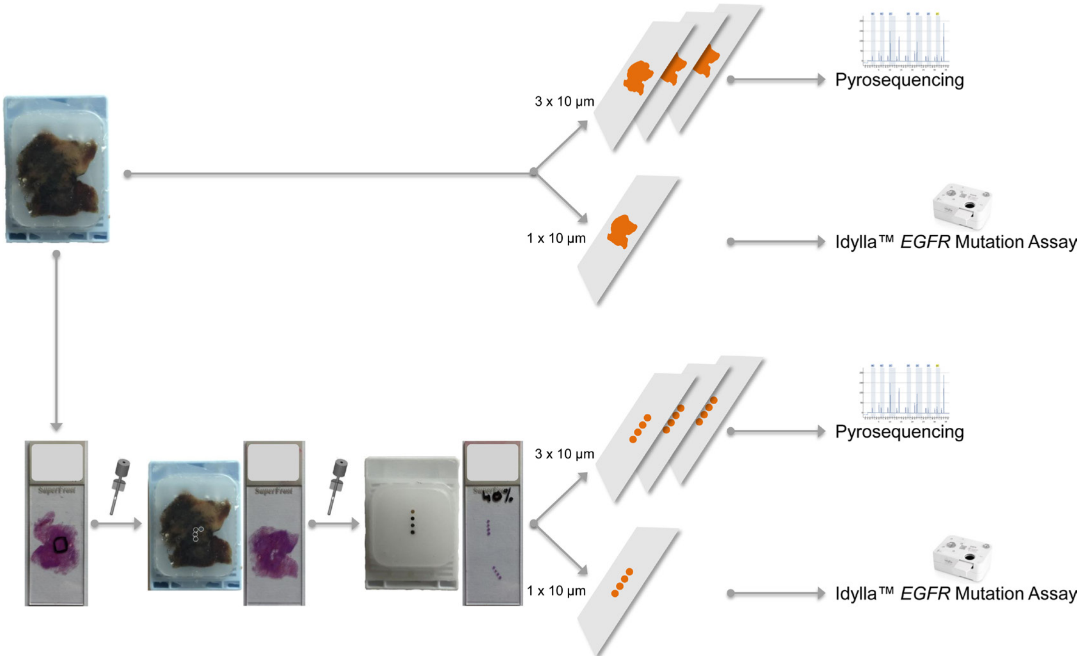

Supplementary Figure 2: The workflow of different analyzed tumor samples.

Supplementary Table 1: Idylla™ EGFR mutation assay performance on whole tissue sections and TMA biopsy cores. See Supplementary_Table_1

Supplementary Table 2: *EGFR* mutations tested for both technologies. See Supplementary_ Table_2
